# High-Precision Measurement of Sea Surface Temperature with Integrated Infrared Thermometer

**DOI:** 10.3390/s22051872

**Published:** 2022-02-27

**Authors:** Kailin Zhang, Xinyu Wang

**Affiliations:** College of Marine Technology, Faculty of Information Science and Engineering, Ocean University of China, Qingdao 266005, China; wxy5615@stu.ouc.edu.cn

**Keywords:** sea surface temperature, radiometer, infrared radiation, Planck formula, infrared thermometer

## Abstract

The sea surface temperature (SST) is a crucial parameter system in climate monitoring. Satellite remote sensing is currently the most common approach for measuring long-term and large-area sea surface temperatures. The SST data measured by the satellite radiometer include the sea surface skin temperature (SSTskin) at a depth of approximately 10 μm. Satellite remote sensing measurement data must be compared and validated with on-site measured data. There are various solutions for on-site measuring instruments; the essential components are usually infrared radiation sensors with radiation output. This paper uses an ordinary integrated infrared thermometer without a radiation output function to remotely measure the sea surface temperature to achieve a high-precision measurement. The scheme of integrating infrared thermometers to measure the sea surface temperature is investigated in this paper. Based on Planck’s formula, the bidirectional conversion relationship between temperature and radiation in a certain band is established. The experimental system introduced in this paper uses an integrated infrared thermometer to measure the small blackbody and the target in a cyclic measurement system. We combine it with the sea surface emissivity characteristics and eliminate the influence of sky background radiation on the sea surface to obtain the actual amount of radiation on the sea surface, from which we obtain the actual radiation amount on the sea surface. Accurate SST can be calculated from the actual amount of radiation at the sea surface. The temperature measurement accuracy can reach 0.1 K, allowing it to meet on-site temperature measurement requirements, as well as the comparison measurement requirements confirmed by satellite remote sensing on-site data. There are relatively few products available for sensors with a temperature measurement accuracy of 0.1 K on the market, and temperature measurement equipment with a temperature measurement accuracy of 0.1 K is relatively expensive. Cost is one of the important factors to consider when using in bulk, especially as global warming increases the need for ocean monitoring. The scheme proposed in this paper is beneficial to reduce the volume and weight of measuring instruments, reduce the cost, and promote the large-scale combined application of sea surface temperature change monitoring.

## 1. Introduction

To meet the requirements for the accuracy of satellite SST data in climate data records, high-precision SST on-site measurement data are required to verify the satellite data [1,2,3]. Due to the heat flux exchange at the air–sea interface, the vertical distribution of water temperature in the upper ocean is uneven. The seawater skin temperature refers to the temperature of the thin water layer of 0–500 um. The measurement of seawater temperature by sensors, such as buoys, will damage the water body and cannot accurately measure the seawater skin temperature. Therefore, infrared radiometers are generally used for on-site measurements [1]. The most representative infrared thermometers used in the world to measure sea surface temperature include the infrared sea surface temperature autonomous radiometer (ISAR), calibrated infrared in situ measurement (CIRMIS), scanning infrared sea surface temperature radiometer (SISTeR), marine-atmospheric emitted radiance interferometer (M-AERI), and so on [4,5,6,7,8]. ISAR uses a KT15 radiometer to detect blackbody radiation at different temperatures through a rotating plane mirror. A linear relationship is fit between the radiation and the value measured by the radiation sensor inside the blackbody, and the radiometer is calibrated to obtain the true sea surface temperature by this linear relationship [9,10]. CIRMIS uses two independent KT11 infrared radiometers to get the radiation intensity of the sky and the sea and uses a dynamically set high-precision blackbody to the correct temperature [11,12]. The core of M-AERI is the FTIR interferometer. The calibration consists of two parts: spectrum and radiation. Spectral calibration provides a degree of reliability in the characteristic position in the measured infrared spectrum; radiation calibration means that the FTIR spectroradiometer can be calibrated by using two blackbody targets at known temperatures [2,13].

Most of the above-mentioned radiometer remote sensing temperature measurement instruments are radiation measurement sensors with radiation output. The main feature common to all is that the temperature sensor takes measurements related to radiation. Subsequent circuits and firmware programs use this radiation measurement as the main parameter to invert the target temperature. These sensors are characterized by a complex structure, large volume, and relatively high price. The measurement scheme discussed in this paper adopts a different from the traditional scheme, using an inexpensive integrated infrared temperature sensor instead of an infrared radiometer. This integrated infrared temperature sensor can only output the measured temperature, but it has a simple structure, a small size, and a relatively affordable price. For the installation of sea surface temperature measurement equipment on every scientific buoy, offshore oil platform, and long-distance freighter, cost is a factor to consider. If the commercially available integrated infrared temperature measurement sensor is directly aimed at the sea surface, temperature measurement can also be carried out, but the accuracy is not adequate. Using an integrated infrared temperature sensor instead of an infrared radiometer to measure high precision sea surface temperature, compared with the traditional radiometer temperature measurement scheme, requires a series of calculations. The specific method is to obtain the radiation corresponding to the temperature of each measurement target through calculation. Combined with other radiation relationships that affect the sea surface temperature, the quantity of radiation relating to sea surface temperature is obtained, and the sea surface temperature value corresponding to the radiation amount is calculated. The Stefan–Boltzmann law integrates Planck’s formula in all wavebands to obtain the relationship between temperature and radiation amount in all wavebands. Since the wavelength range of the integrated infrared temperature sensor is not the whole band, this paper theoretically deduces the bidirectional relationship between temperature and radiation within the band range of the infrared thermometer. Through theoretical analysis and experimental verification, the real-time blackbody calibration scheme is adopted to improve the measurement accuracy of the integrated temperature sensor, from 0.5 to 0.1 K, which can better complete the measurement of SST. This precision can meet the temperature measurement requirements of environmental scientists, as well as satellite remote sensing field data verification requirements.

## 2. Materials and Methods

The scheme in this paper uses the infrared temperature sensor MLX90614 and the pyroelectric integrated infrared radiometer KT19 to take seawater temperature and sky temperature, respectively. We established a bidirectional conversion relationship between temperature and radiation, and calculated the radiation corresponding to the temperature. According to the principle of sea surface temperature measurement, the radiation of seawater surface temperature is obtained, and finally the seawater surface temperature corresponding to the radiation is calculated.

### 2.1. Establish the Conversion Relationship between Temperature and Radiation Quantity

The Planck formula proposed the law of blackbody radiation: at any temperature, the relationship between the emissivity of the blackbody electromagnetic radiation and the wavelength of electromagnetic radiation is

(1) $$\begin{array}{c} B\left(\lambda ,T\right)=\frac{2hc^{2}}{\lambda^{5}}\frac{1}{e^{\mathrm{hc}/\lambda kT}-1} \end{array}$$

According to the equation, λ is the wavelength of electromagnetic waves, the speed of light in vacuum $c=2.998\times 108\;{\mathrm{ms}}^{-1}$, Planck constant $h=6.626\times 10^{-34}\;J\cdot s$, Boltzmann constant $k=1.381\times 10^{-23}\;J\cdot K^{-1}$, and T is the absolute temperature in Kelvin (K). The unit of B(λ,T) is $W/m^{3}$.

When $c_{1}=2\;{\mathrm{hc}}^{2}=3.7415\times 10^{-16}\;W\cdot m^{2}$, $c_{2}=\mathrm{hc}/k=1.4388\times 10^{-2}\;\mathrm{mK}$, the formula can be written

(2) $$\begin{array}{c} B\left(\lambda ,T\right)=\frac{c_{1}}{\lambda^{5}}\frac{1}{e^{c_{2}/\lambda T}-1} \end{array}$$

Planck’s formula represents the amount of blackbody radiation at any single wavelength at any temperature. By integrating the Planck formula with wavelength λ1 and λ2 as upper and lower limits, the blackbody radiation amount at any temperature can be obtained; thus, the blackbody radiation amount represented by any temperature of the target measured by the infrared thermometer can be directly obtained. The integral of the fixed band range can be obtained as follows:

(3) $$\begin{array}{c} M=\int_{\lambda 1}^{\lambda 2}B(\lambda ,T)d\lambda =\int_{\lambda 1}^{\lambda 2}\frac{c_{1}}{\lambda^{5}}\frac{1}{e^{c_{2}/\lambda T}-1}d\lambda \end{array}$$

In the above formula, $x=c_{2}/$$\lambda_{1}T$, $\mathrm{dx}=-c_{2}/$$\lambda_{2}$*T* dλ

(4) $$\begin{array}{c} M=\int_{x_{1}}^{x_{2}}\frac{c_{1}}{\lambda_{5}}.\frac{\lambda^{2}T}{c_{2}}.\frac{1}{e^{x}-1}dx=\frac{c_{1}}{{c_{2}}^{4}}T^{4}\int_{x1}^{x2}\frac{x^{3}}{e^{x}-1}dx \end{array}$$

The denominator of an integral part of Equation (4) is expanded by the following series

(5) $$\begin{array}{c} \frac{1}{e^{x}-1}=\frac{e^{-x}}{1-e^{-x}}=e^{-x}\left(1+e^{-x}+e^{-2x}+....+e^{-nx}\right)=\sum_{n=1}^{\infty }e^{-nx} \end{array}$$

Rearrange

(6) $$\begin{array}{c} M=\frac{c_{1}}{{c_{2}}^{4}}T^{4}\int_{x1}^{x2}x^{3}\sum_{n=1}^{\infty }e^{-nx}dx=\frac{c_{1}}{{c_{2}}^{4}}T^{4}\sum_{n=1}^{\infty }\int_{x1}^{x2}x^{3}e^{-nx}dx \end{array}$$

In this case, the integration by parts method is used to solve the definite integral

(7) $$\begin{array}{c} {\left.M=\frac{c_{1}}{{c_{2}}^{4}}T^{4}\sum_{n=1}^{\infty }\left(-\frac{1}{n}x^{3}e^{-nx}-\frac{3}{n^{2}}x^{2}e^{-nx}-\frac{6}{n^{3}}xe^{-nx}-\frac{6}{n^{4}}e^{-nx}\right)\right|}_{x1}^{x2} \end{array}$$

At this time, ×1 and ×2 are not complicated and are variables containing temperature. The wavelength range of the infrared thermometer used in this paper is 5.5–14 μm, that is, $x_{1}=c_{2}/$$\lambda_{1}T$, $x_{2}=c_{2}/$$\lambda_{2}T$.

**Figure 1:**
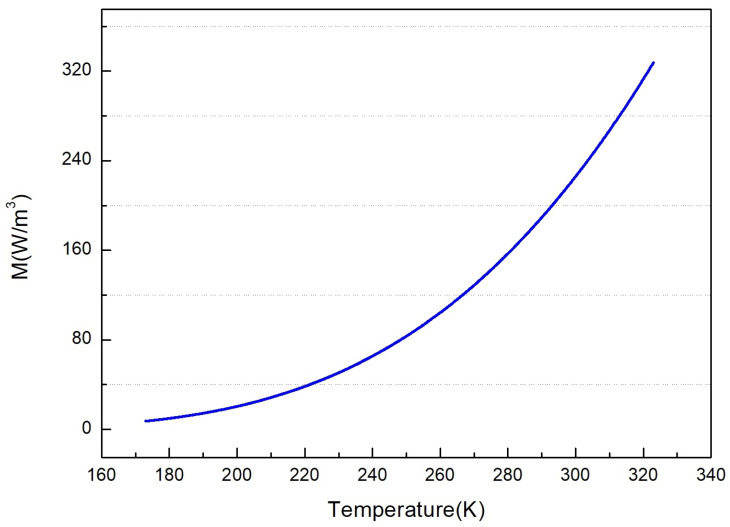
Theoretical relationship between temperature and radiation quantity.

Combined with the temperature acquisition range of this paper, which is about 173–323 K, calculate the amount of the radiation corresponding to the temperature directly output by the infrared thermometer. The calculation relationship is shown in Figure 1. Since the temperature acquisition range in this paper is between 173 and 323 K, only the radiation amount corresponding to the temperature in this range is output, as shown in the figure. The above is the first step in establishing the conversion relationship between temperature and radiation. The Equation (7) applies to any type of sensor, and the formula does not limit the temperature range.

The second step is to fit the functional relationship between temperature and radiation. The functional relationship fitted in this paper will be used on embedded devices. Considering the processing capability of the embedded system, the relationship between temperature and radiation is fitted, and the accuracy of the fitted relationship is within ±0.005 K. The relationship between radiation and temperature is as follows:

(8) $$\begin{array}{c} B=LB\left(T\right)=a_{1}T^{5}+a_{2}T^{4}+a_{3}T^{3}+a_{4}T^{2}+a_{5}T+a_{6} \end{array}$$

where the coefficients of the fitted relationship are as follows:

$a_{1}=-2.38712242\times 10^{-10}$; $a_{2}=2.74845748\times 10^{-7}$; $a_{3}=-7.7567487\times 10^{-5}$;

$a_{4}=8.46359184\times 10^{-3}$; $a_{5}=-2.68039688\times 10^{-1}$; $a_{6}=-7.1712316$.

The relationship between temperature and radiation is bidirectional. This paper will also use the relationship with the known radiation to find the temperature, as follows:

(9) $$\begin{array}{c} T=LB^{-1}\left(B\right)=b_{1}\log{\left(B\right)}^{5}+b_{2}\log{\left(B\right)}^{4}+b_{3}\log{\left(B\right)}^{3}+b_{4}\log{\left(B\right)}^{2}+b_{5}\log\left(B\right)+b6 \end{array}$$

where the coefficients of the fitted relationship are as follows:

$b_{1}=1.09098611\times 10^{-2}$; $b_{2}=-1.31423943\times 10^{-1}$; $b_{3}=1.00368321$;

$b_{4}=-1.18568103$; $b_{5}=1.92352834\times 10^{1}$; $b_{6}=1.33128573\times 10^{2}$.

**Figure 2:**
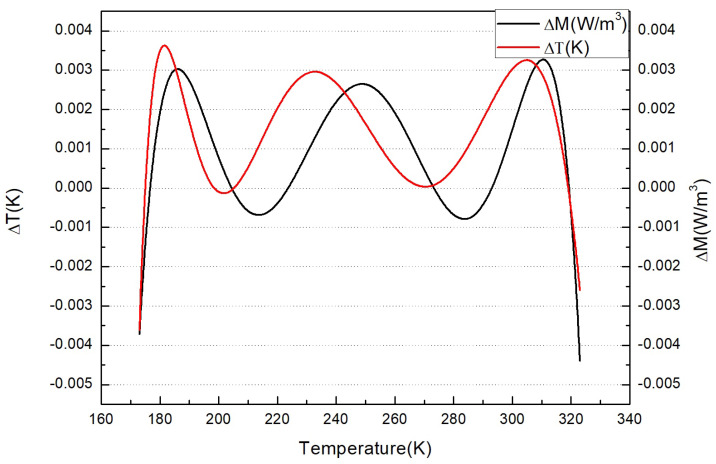
Fitting function and algorithm comparison.

Furthermore, *B* = *LB*(*T*) represents the function relation from temperature to radiation amount, and the fitting function and algorithm error curve are shown in Figure 2. $T={\mathrm{LB}}^{-1}\left(B\right)$ represents the inverse operation function relation from the radiation amount to the temperature, and the deviation curve of the two is shown in the Figure 2.

The data directly detected by the integrated infrared thermometer are the temperature values. The corresponding radiation value is obtained through the above-fitting function. Combined with the relationship between the radiation of each part of the seawater surface described in the following chapters, the real seawater surface radiation amount is obtained. Finally, the inverse operation function relationship is used to calculate the sea surface temperature.

### 2.2. Principle of Measuring Sea Surface Temperature

When the temperature measurement equipment deployed on the sea or on the mobile platform measures the sea surface temperature, the observed temperature not only includes the actual sea surface temperature, but is also affected by other factors, such as the sky and air [10]. The radiation amount of an ideal blackbody can be calculated directly by Planck’s formula. The actual radiation amount of an object should be multiplied by the emissivity value of this object by the blackbody radiation at the equivalent temperature, the effective emission radiation, which is the value when applied to seawater. Furthermore, the emissivity is known as the water-leaving radiance [14].

(10) $$\begin{array}{c} L_{\lambda }\left(T\right)=\varepsilon \times B_{\lambda }\left(T\right) \end{array}$$

**Figure 3:**
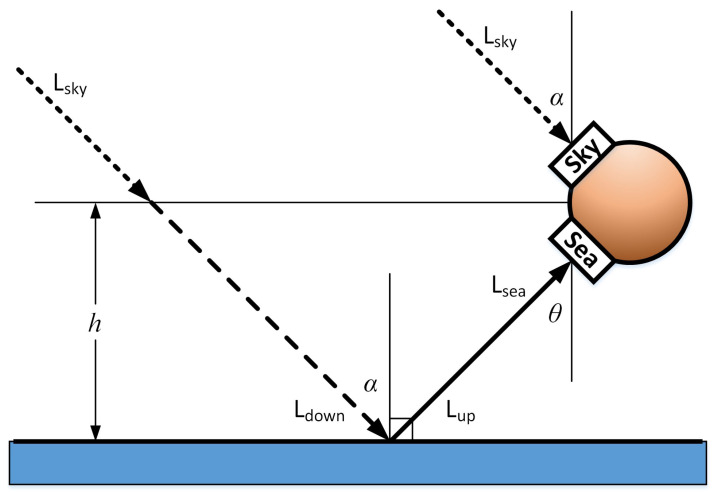
Radiation component of SST [8].

It can be seen from Figure 3 that the single-wavelength downlink radiation from the sea surface mainly comes from the sky, and a small part comes from the air. When the transmittance along the atmospheric path is close to 1, the air part can be ignored. Most of the single-wavelength upward radiation passing through the sea surface comes from the seawater surface, and the other part comes from the reflection of the downward radiation from the sea surface. When infrared temperature measurement equipment observes seawater, the single-wavelength radiation reaching the observation equipment is

(11) $$\begin{array}{c} L_{\mathrm{sea}}\left(\lambda \right)=\varepsilon \left(\lambda ,\theta \right)B\left({\mathrm{SST}}_{\mathrm{skin}},\lambda \right)+\left[1-\varepsilon \left(\lambda ,\theta \right)\right]L_{\mathrm{sky}} \end{array}$$

*L*_sky_ is the radiation amount of a single wavelength when infrared temperature measuring equipment observes the sky. B (SST_skin_, λ) is the true amount of radiation at sea surface temperature [10,14]. The wavelength range of the infrared detection equipment used in this paper is 5.5–14 um, so all of the radiation reaching the equipment is integral of the radiation of a single wavelength in the waveband range.

(12) $$\begin{array}{c} S_{\mathrm{sea}}=\int_{\lambda 1}^{\lambda 2}L_{\mathrm{sea}}\left(\lambda \right)d\lambda =\int_{\lambda 1}^{\lambda 2}\varepsilon \left(\lambda ,\theta \right)B\left({\mathrm{SST}}_{\mathrm{skin}},\lambda \right)+\left[1-\varepsilon \left(\lambda ,\theta \right)\right]L_{\mathrm{sky}}d\lambda \end{array}$$

where $\lambda_{1}$ = 5.5 um, $\lambda_{2}$ = 14 um. In the narrow band, 5.5–14 um, the wavelength changes slowly, and ϵ(λ,θ) can be approximated as ϵ(θ) [10]

(13) $$\begin{array}{c} S_{\mathrm{sea}}=\varepsilon \left(\theta \right)\int_{\lambda 1}^{\lambda 2}B\left({\mathrm{SST}}_{\mathrm{skin}},\lambda \right)d\lambda +\left[1-\varepsilon \left(\theta \right)\right]S_{\mathrm{sky}} \end{array}$$

where *S*_sky_ is the total radiation reaching the equipment when the equipment observes the sky. SST can be obtained by the conversion formula

(14) $$\begin{array}{c} \int_{\lambda 1}^{\lambda 2}B\left({\mathrm{SST}}_{\mathrm{skin}},\lambda \right)d\lambda =\frac{S_{\mathrm{sea}}\left(\lambda \right)-\left[1-\varepsilon \left(\theta \right)\right]S_{\mathrm{sky}}}{\varepsilon (\theta )} \end{array}$$

It can be seen intuitively from the formula that, for the temperature measuring equipment, to measure the sea surface temperature accurately, it is necessary to obtain the radiation measurement values of sea surface radiation and atmospheric radiation. For the integrated infrared thermometer used in this paper, the measured output value is the temperature value, which needs to be converted in combination with the fitting function mentioned above. The actual output signal of the integrated infrared thermometer observed on the sea surface is *T*_sea_. According to the relationship between temperature and radiation amount, *S*_sea_(λ) = *LB*(*T*_sea_) represents the radiation amount measured by the temperature measuring equipment within the band range of 5.5–14 μm. The output signal observed towards the sky is *T*_sky_, *S*_sky_(λ) = *LB*(*T*_sky_), which is convenient for further calculation. After substitution, the formula can be written:

(15) $$\begin{array}{c} S_{{\mathrm{SST}}_{\mathrm{skin}}}=\frac{LB\left(T_{\mathrm{sea}}\right)-\left(1-\varepsilon \left(\theta \right)\right)LB\left(T_{\mathrm{sky}}\right)}{\varepsilon (\theta )} \end{array}$$

The actual sea surface temperature is obtained from the inverse operation function *T* = $LB^{-1}$(*B*).

(16) $$\begin{array}{c} {\mathrm{SST}}_{\mathrm{skin}}=LB^{-1}\left(S_{{\mathrm{SST}}_{\mathrm{skin}}}\right)=LB^{-1}\left[\frac{LB\left(T_{\mathrm{sea}}\right)-\left[1-\varepsilon (\theta )\right]LB\left(T_{\mathrm{sky}}\right)}{\varepsilon (\theta )}\right] \end{array}$$

The above is the basic principle of using an integrated infrared thermometer to measure sea surface temperature instead of other equipment.

## 3. Experiments and Performance Testing

This work used the MLX90614 infrared thermometer, which integrates the infrared induction thermopile detector chip and the signal processing dedicated integrated chip. The official information of the MLX90614 infrared thermometer states that the accuracy of this thermometer reaches 0.5 K, and the resolution is 0.02 K [15]. The algorithm part of this article is not limited to the thermometer used in this article, and other types of infrared temperature sensors from other manufacturers can also achieve similar accuracy. When the infrared thermometer measures the target temperature, the measurement results of the instrument will be affected by its factors and the environment. In order to reduce the influence of these factors on the measurement results, high-temperature black bodies and ambient-temperature black bodies are introduced to improve accuracy [16,17].

**Figure 4:**
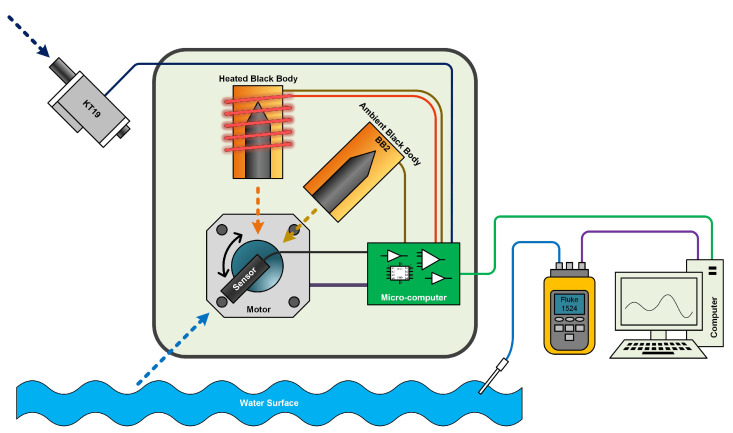
Optical path of equipment: high-temperature blackbody, ambient-temperature blackbody, target water surface, and infrared thermometer.

Based on the temperature measurement principle mentioned above, when observing the surface temperature of seawater, it is necessary to obtain the temperature of the seawater and the sky. Errors can be caused by rapid changes in atmospheric radiation due to different types and heights of clouds and different radiation temperatures, so ideally both sea and sky temperatures should be obtained simultaneously, or the time difference between sea and sky measurements must be small [18]. As a solution to this problem, this paper selects two devices to measure sea surface and the sky almost simultaneously, an integrated infrared thermometer to measure sea surface temperature, and a pyroelectric integrated infrared radiometer KT19 to measure sky temperature(see Figure 4). The specific process entails: using the embedded system to control the rotation of the motor equipped with the infrared thermometer, and each time the infrared thermometer must be aligned with the ambient-temperature blackbody, the high-temperature blackbody, and the water surface to measure the temperature. Immediately after measuring the water surface temperature, an instruction is sent to KT19 to obtain the present temperature of the sky, to meet the small measurement time difference between the sea and the sky. The whole process takes approximately 7400 ms. First, the directly obtained temperature is converted into radiation through the relationship between the temperature and the radiation amount derived above. Combined with the relationship with the seawater surface, the radiation amount of the seawater surface is obtained, and is then the radiation amount is converted into temperature through the relationship between radiation amount and temperature. The real-time temperature can be sent to the computer for viewing through the RS232 external serial port, or stored on the internal SD card to complete data acquisition.

### 3.1. Improvement of Performance Test Experiment

Before formally measuring the water surface, carry out a standard blackbody calibration. Aim the device to a standard blackbody radiation source, which is a standard blackbody imported from Canada LR TECH INC., model BB-ASSIST II. The parameter accuracies of the standard blackbody official data are as follows: the radiation temperature stability is less than ±0.005 K, and the temperature display accuracy is 0.1 °C. In the experiment, the standard blackbody is set to 25 °C (296.15 K), and the measurement is started after the temperature stable. During the measurement, the temperature of the standard blackbody has been keptat 25.02 °C (296.17 K). The specific process uses the embedded system to control the rotation of the motor equipped with the infrared thermometer, and each time the infrared thermometer needs to be aligned with the ambient-temperature blackbody, the blackbody, and the standard blackbody to take the temperature, correct the accuracy of infrared thermometers in real-time and eliminate the influence of environmental factors.

**Figure 5:**
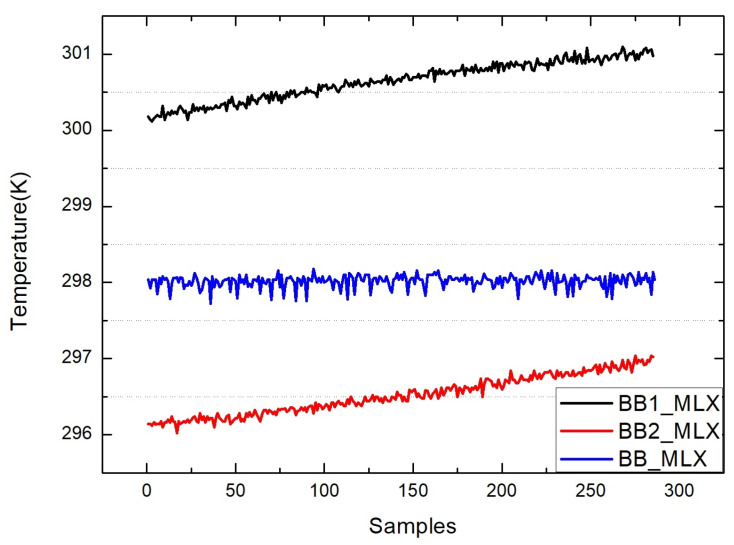
Measurement results of an MLX90614 infrared thermometer directly aimed at a high-temperature blackbody (BB1_MLX), ambient-temperature blackbody (BB2_MLX), and standard blackbody (BB_MLX).

**Figure 6:**
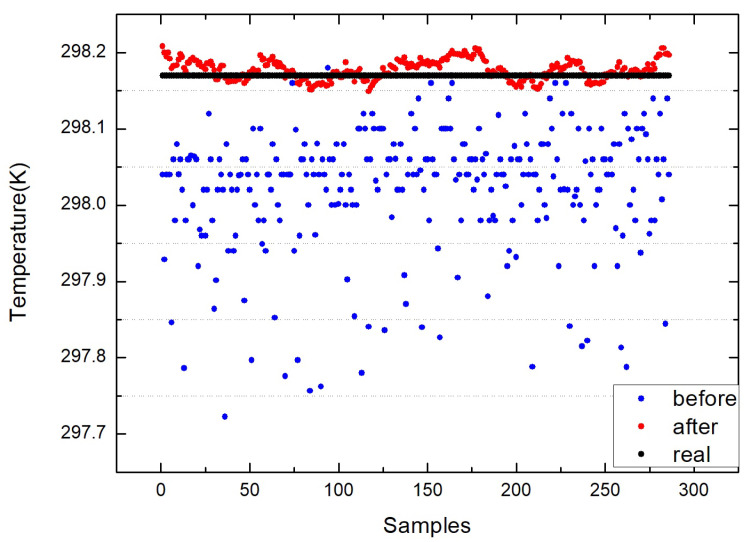
Output result (before) of directly aligning the blackbody, the final output result (after) after aligning the standard blackbody after algorithm correction, and the actual temperature (real) of the standard blackbody.

**Figure 7:**
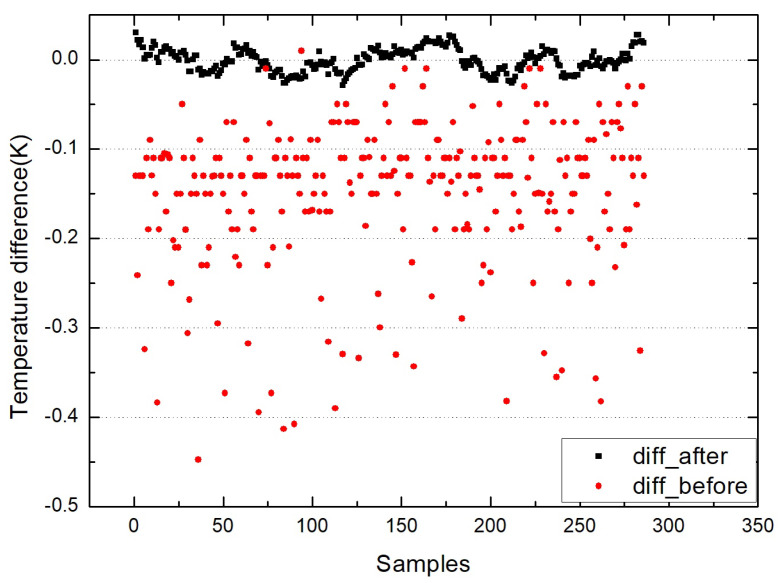
Deviation of the MLX90614 infrared thermometer before correction (diff_before) and the deviation after correction (diff_after).

The figures above show the output of all measurement data when using standard blackbody calibration. Figure 5 shows the measurement results of an MLX90614 infrared thermometer directly aimed at high-temperature blackbody (BB1_MLX), the ambient-temperature blackbody (BB2_MLX), and the standard blackbody (BB_MLX). These data are the direct output result of MLX90614, which is not processed by the algorithm in this paper, the bias is −0.150; the standard deviation is 0.083. Figure 6 shows the output result of direct alignment to the blackbody (before), the final output result after algorithm correction (after), and the actual temperature of the standard blackbody (real). The corrected result is clearly closer to the actual temperature and more stable. Figure 7 shows that the deviation of the MLX90614 infrared thermometer directly measures the standard blackbody from the actual temperature and the deviation from the actual temperature after algorithm correction. The bias before algorithm processing is −0.150; the standard deviation is 0.083. The deviation from the actual temperature after treatment was within ±0.035 K, the bias was 0.007, and the standard deviation was 0.013. By calculating the radiation through an algorithm, the thermometer with the original accuracy of 0.5 K can be increased from 0.5 to 0.035 K.

### 3.2. Observing the Water Surface Experiment

A circulating constant temperature water tank was used to simulate the seawater surface in the laboratory for theoretical verification. The constant temperature water tank was placed in the natural environment so that the water surface was affected by the radiation from the sky. After the temperature stabilizes, the angle of the integrated infrared thermometer to observe the water surface is set to 45 degrees. The equipment used to measure the sky temperature was KT19, and the measurement angle was also set to 45 degrees. At the same time, the high-precision thermometer FLUKE1524 was used to measure the water surface, in order to obtain the reference temperature of the water surface.

**Figure 8:**
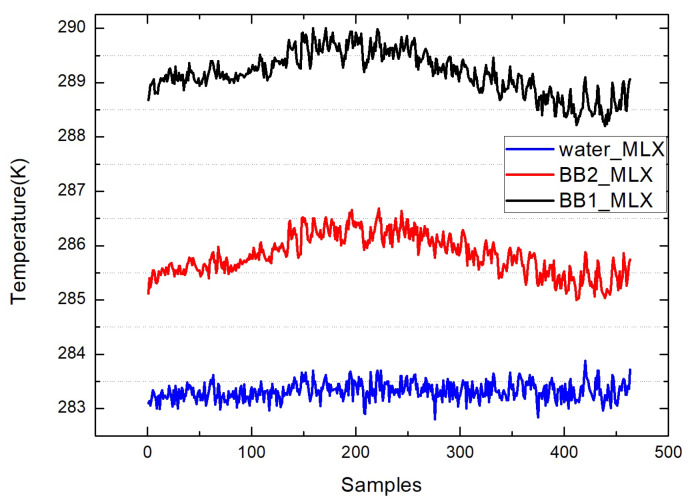
The infrared thermometer directly measures the output values of the high-temperature blackbody (BB1_MLX), ambient-temperature blackbody (BB2_MLX), and water surface (water_MLX).

**Figure 9:**
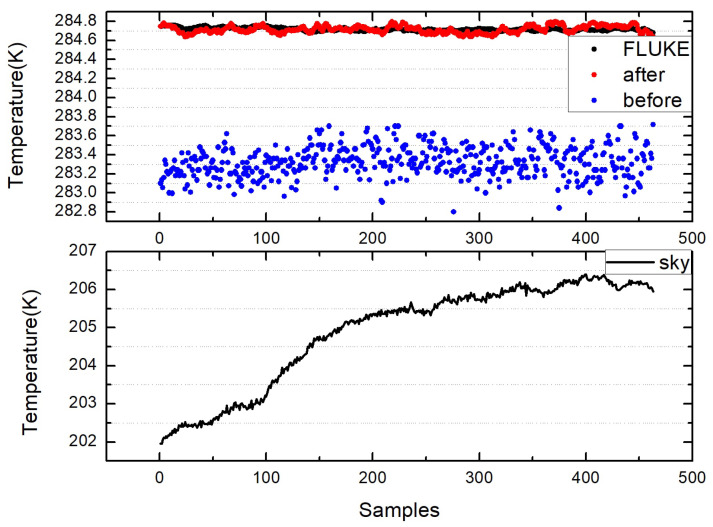
Measured value of the infrared thermometer on the water surface (before), the output water surface temperature value after the algorithm in this paper (after), the measured value of the water surface temperature from FLUKE (FLUKE), and the measured value of the sky from KT19 (sky).

**Figure 10:**
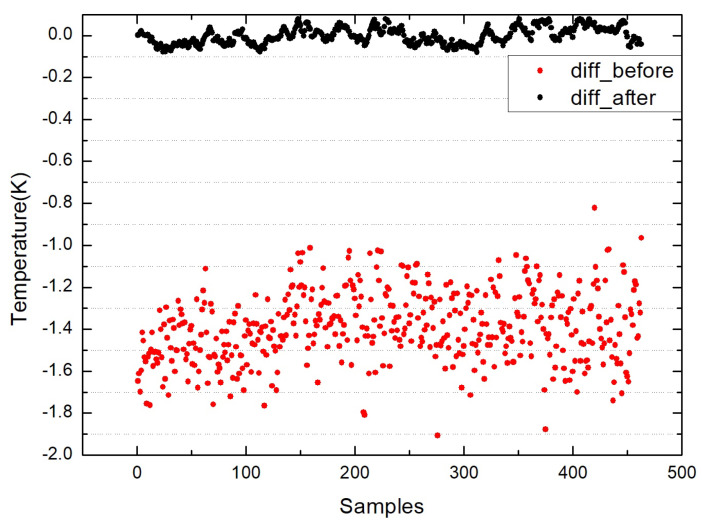
Deviation before correction of the infrared thermometer (diff_before) and after correction (diff_after) shows the comparison between the output water surface temperature value and the measured value of FLUKE1524 and the deviation value of the two after the derivation of the algorithm in this paper.

The above figures show the output of all measurement data when the laboratory uses a circulating constant temperature water tank to simulate the seawater surface for theoretical verification. Figure 8 shows the measurement results of the MLX90614 infrared thermometer directly aimed at the high-temperature blackbody (BB1_MLX), the ambient-temperature blackbody (BB2_MLX), and the water surface (water_MLX). These data are the direct output result of MLX90614, which is not processed by the algorithm in this paper. Figure 9 shows the measured value of the water surface by the infrared thermometer (before), the water surface temperature (after), and the water surface temperature measured by FLUKE. (FLUKE) and KT19 measurements of the sky (sky). The corrected result is closer to the actual temperature and more stable. Figure 10 shows the deviation between the direct measurement of the water surface and the measured value of FLUKE1524 by an MLX90614 infrared thermometer, and the deviation from the measured value of FLUKE1524 after algorithm correction. The bias before algorithm processing is −1.393, and the standard deviation is 0.169; after processing, the bias is 0.002, and the standard deviation is 0.039. By calculating the radiation through the algorithm, the thermometer accuracy can be increased to 0.035 K. Compared with many experiments, the deviation of the temperature value does not exceed ±0.1 K.

## 4. Discussion and Conclusions

This paper focuses on integrating infrared thermometers to measure sea surface temperature. Based on Planck’s formula, the bidirectional relationship between temperature and radiation in the band range is established. The pyroelectric integrated infrared thermometer in the traditional solution can be replaced by an integrated infrared thermometer that only outputs temperature. The temperature measured by the integrated infrared thermometer is converted from the temperature-radiation amount relationship, and the radiation amount is calculated.

In this paper, we built an experimental system to test and verify the above temperature measurement scheme, using the MLX90614 integrated infrared temperature measurement sensor and two small black bodies with different temperatures. The small blackbody corrects the sensor in real time and adds a motor with a controllable rotational position to switch the measurement angle of the sensor. First, the experimental system conducts temperature measurement experiments on the BB-ASSIST II standard blackbody. The temperature measurement bias of the integrated temperature sensor without real-time calibration to the standard blackbody is −0.150, and the standard deviation is 0.083. After using the measurement method proposed in this paper, the temperature measurement bias of the measured standard blackbody is 0.007, and the standard deviation is 0.013. Then, the water temperature measurement experiment of the circulating constant temperature water tank under the natural sky radiation environment is carried out. The radiation amount is calculated after using the measurement method proposed in this paper, combined with the relationship between water surface radiation, sky radiation, and water temperature. Finally, the temperature at 10 um of the sea surface is obtained from the radiation–temperature relationship; the temperature measurement bias of the water body surface is 0.002, and the standard deviation is 0.039. It can be seen that the scheme in this paper has a significant effect on improving the measurement accuracy of the integrated temperature sensor.

The main shortcoming of this paper is that the whole system does not use all infrared temperature measurement sensors, and the sensor Heitronics KT19 is used alone to measure the radiation of the sky. The MLX90614 infrared temperature measurement sensor has a large temperature measurement deviation for the sky and cannot accurately measure the low-temperature background radiation of the sky. In the following work, a suitable integrated temperature sensor will be selected to replace the KT19, to obtain smaller device volume and better cost performance.

## Data Availability

The data supporting this study are provided within this paper.
